# Generalized Negative Reciprocity in the Dictator Game – How to Interrupt the Chain of Unfairness

**DOI:** 10.1038/srep22316

**Published:** 2016-02-29

**Authors:** Sabrina Strang, Xenia Grote, Katarina Kuss, Soyoung Q. Park, Bernd Weber

**Affiliations:** 1 Department of Psychology, University of Lübeck, Lübeck, Germany; 2 Center for Economics and Neuroscience, University of Bonn, Bonn, Germany; 3 Department of Psychiatry, University Hospital Bonn, Bonn, Germany; 4 Department of Epileptology, University Hospital Bonn, Bonn, Germany

## Abstract

Humans are tremendously sensitive to unfairness. Unfairness provokes strong negative emotional reactions and influences our subsequent decision making. These decisions might not only have consequences for ourselves and the person who treated us unfairly but can even transmit to innocent third persons – a phenomenon that has been referred to as generalized negative reciprocity. In this study we aimed to investigate whether regulation of emotions can interrupt this chain of unfairness. Real allocations in a dictator game were used to create unfair situations. Three different regulation strategies, namely writing a message to the dictator who made an unfair offer, either forwarded or not forwarded, describing a neutral picture and a control condition in which subjects just had to wait for three minutes, were then tested on their ability to influence the elicited emotions. Subsequently participants were asked to allocate money between themselves and a third person. We show that writing a message which is forwarded to the unfair actor is an effective emotion regulation strategy and that those participants who regulated their emotions successfully by writing a message made higher allocations to a third person. Thus, using message writing as an emotion regulation strategy can interrupt the chain of unfairness.

Across cultures humans have a strong preference for fairness^1^,^2^. Violations of fairness provoke negative emotional responses^3–5^. Furthermore, perceived unfairness can influence related decision making. People who were treated unfairly tend to pay this back by treating the same person unfairly as well, a phenomenon called negative reciprocity^6,7^. However, the consequences of unfair behavior seem to have an even larger extent. People do not only behave unfairly against the person who treated themselves unfairly, but do also forward this behavior towards uninvolved third persons (generalized negative reciprocity^8^,^9^). Unfairness can thus spread easily and once the chain of unfairness is activated, it is difficult to interrupt.

If negative emotions are the underlying factor driving general negative reciprocity, effective emotion regulation should lead to a decrease in general negative reciprocity. For example, the chance to express emotions in another way as by punishing the offender was shown to influence rejection rates in an ultimatum game. Here, participants who had the opportunity to express their emotions via a message to the proposer showed lower rejection rates^10^. A common method of emotion regulation is reappraisal, in which subjects are asked to mentally reframe and reanalyze the context^11^,^12^. Reappraisal has been shown to decrease rejection rates in the ultimatum game^13^,^14^.

However, in these studies emotions in the ultimatum game were either not measured explicitly or did not change after regulation. Measuring both emotions and rejection rates bears the risk of an interaction between the two variables. Moreover, assuming that message writing alters rejection rates via emotion expression is a little farfetched, based on this evidence alone. Messages consist of several components besides emotion expression, for example writing in general, additional content elements and forwarding of the message. Which of these components drive the effect of altered rejection rates remains unclear.

Thus, participants show generalized negative reciprocity: they transmit unfairness to innocent third persons^8^. Further, emotion regulation strategies were shown to decrease direct negative reciprocity in the ultimatum game^13^,^14^. The following questions remain open: 1) Does message writing as an emotion regulation strategy effectively alter negative emotions due to unfair social situations? And if so, which component of the writing process drives this effect? 2) Does an effective emotion regulation strategy decrease *general* negative reciprocity and thereby interrupting the chain of unfairness? Answering the first question will extend our knowledge of social interactions. Since unfairness is a hazard for social interactions answering the second question will contribute in improving the quality of those.

We conducted two studies investigating whether emotion regulation can decrease general negative reciprocity. In study 1 unequal allocations in a dictator game creating unfair situations were tested on their effect on affective responses. Three emotion regulation strategies were then tested on their ability to regulate the provoked emotional reactions. Affective responses were measured using the pleasure subscale of the Self-Assessment Manikin^15^. This subscale measures affective responses on a scale ranging from happy to unhappy. In study 2 participants were additionally asked to allocate money between themselves and a third person in order to measure generalized negative reciprocity (Fig. 1A). We hypothesized that 1) writing a message to the dictator who made the unfair allocation will successfully regulate emotions and that 2) as a result participants in the message writing condition will make higher allocations to an unrelated third person.

## Study 1 Methods

In study 1, 237 (*M_age_* = 22.49 years, *SD* = 4.15) participants took part. The study was conducted at the BonnEconLab at the University of Bonn using z-Tree 20. The study meets all standards for ethical treatment of human subjects in experiments at the BonnEconLab and was approved by the review board of the department of economics. Participants were recruited with the software hroot^16^. Only female participants were invited because based on previous studies, we expected higher emotional reactivity to negative stimuli in women^17^,^18^. Since participants needed to show negative affective responses in order to regulate them, we concentrated on women as participants. Participants were randomly allocated to the different conditions and written consent was given according to the Declaration of Helsinki.

Participants were receivers in a dictator game. First, all participants were asked to indicate how happy they felt (i.e. *baseline Happiness*). They then received an allocation from a dictator, who made this decision in a previous session. In all conditions 17% of the participants received a fair allocation (12.50 €/12.50 €) and 83% an unfair allocation (20 €/5 €). This distribution reflected the allocations made by a group of dictators (*N* = 24) who participated in the experiment in a previous session. After receiving the allocation participants were again asked to indicate their affective state (*Happiness 1*). In two of the emotion regulation conditions participants were asked to write a message to the dictator who made the allocation. In the first emotion regulation condition (the forwarded condition), participants were told that the dictator will come to the lab to receive the message they wrote. In the second emotion regulation condition (the non-forwarded condition), it was told that the message will not be forwarded to the dictator. In both conditions participants received this information before they wrote the messages. No further instructions about the content of the messages were given. In the third emotion regulation condition participants were asked to write a description about an emotionally neutral picture (IAPS picture No. 7185 19). The control condition consisted of a waiting period of identical length. Each condition took three minutes. Subsequently participants indicated again how happy they felt (*Happiness 2*). The dictators of the previous session had to come to the lab for the second time in order to receive the messages written by the participants in the message forwarded condition.

Finally, 382 additional participants evaluated the content of the messages written in both message writing conditions via an online questionnaire (see S1 for instructions). Each message was evaluated by at least 62 participants. They rated whether the messages contained content elements as expression of emotions, understanding, unfairness criticism, questioning of motive or suggestion for usage. Of all participants, five were randomly selected and won a 10 € Amazon voucher. We further evaluated whether the messages contained welcome words, exclamation marks, emoticons, abusive language and/or a form of address and determined the average number of characters of each message.

## Results and Discussion

Since we were interested in the effect of emotion regulation in unfair situations we focused only on the unfair allocations in the analysis (analysis of fair allocations can be found in S2). In order to test whether unfair allocations decreased happiness ratings we compared ratings before and after participants received the unfair allocations (*baseline Happiness* versus *Happiness 1*). 12 participants had to be excluded from the analysis due to a failure of understanding the procedure. Since the happiness ratings were not normally distributed a Wilcoxon-signed-rank-test for dependent samples was computed to test for differences between *baseline Happiness* ratings and *Happiness 1* ratings. Happiness ratings after receiving the unfair allocation were significantly decreased (*baseline Happiness Mdn* = 6, *Happiness 1 Mdn* = 4, *z* = −10.17, *p* < 0.001, *r* = −0.55, 95% C.I. was estimated by using the Hodges Lehmann estimator [−2.50, −2.00]).

**Figure 1:**
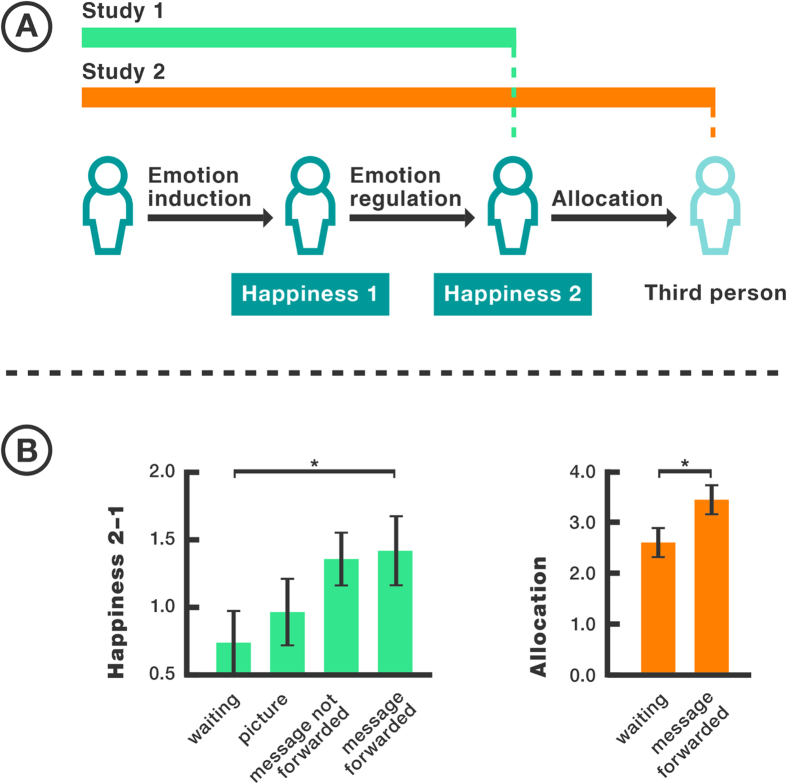
Study designs and results. (**A**) Study design; in study 1 three different emotion regulation strategies were tested, in study 2 participants additionally made allocations to a third person. (**B**) (left) Estimated marginal mean differences between Happiness 2 and 1, happiness significantly increased in the message forwarded condition compared to the control condition. (right) For illustration purposes we indicate mean allocations in the dictator game. Allocations were significantly larger in the message forwarded condition compared to the control condition (* indicates p < 0.05 and the error bars indicate the standard error of mean (SEM)).

To compare the effects of the three different conditions on happiness ratings an ANCOVA was conducted. The difference between *Happiness 1* and *Happiness 2* was used as dependent variable and *baseline Happiness* was included as covariate to control for baseline differences. We observed a significant main effect of condition on the difference between happiness ratings (*F* (3, 157) = 2.84, *p* = 0.04, 95% C.I. [0.284, 1.23], partial *η*^*2*^ = 0.05). The interaction between the condition and baseline rating was not significant (*F* (3, 157) = 2.55, *p* = 0.06). We therefore focused on the main effect of condition. Planned contrasts revealed that message writing with forwarding significantly increased happiness ratings compared to the control condition (*t* = 4.95, *p* = 0.01, 95% C.I. [1.21, 8.68], Cohens’d = 2.62). We did not observe any significant difference between happiness rating in the writing without forwarding compared to the control condition and the picture condition compared to the control condition (writing without forwarding vs. control: *t* = 2.50, *p* = 0.21, picture vs. control: *t* = 1.03, *p* = 0.55; Fig. 1B left). A direct comparison between happiness ratings in the writing with forwarding and writing without forwarding condition yielded no significant difference (*z* = −0.13, *p* = 0.89).

Furthermore, there was no difference in content between forwarded and non-forwarded messages (expression of emotions: *X*^*2*^(60) = 72.27, *p* = 0.13; understanding: *X*^*2*^(52) = 65.18, *p* = 0.10; unfairness criticism: *X*^*2*^(60) = 70.5, *p* = 0.17; questioning of motive: *X*^*2*^(48) = 58.02, *p* = 0.15; suggestion for usage: *X*^*2*^(42) = 53.62, *p* = 0.11). The messages only differed in the frequency of welcome words and exclamation marks used (welcome words: *X*^*2*^(1) = 5.21, *p* = 0.019; exclamation marks: *X*^*2*^(1) = 4.89, *p* = 0.025). Forwarded messages contained more welcome words and exclamation marks.

In order to test, whether the content of the messages was related to changes in happiness ratings a correlation between the frequencies with which messages (with forwarding and without forwarding) were rated to contain specific content elements (expression of emotions, understanding, unfairness criticism, questioning of motive or suggestion for usage) and the difference between *Happiness 1* and *Happiness 2* was conducted. We observed a significant correlation between emotion expression and change in happiness ratings (*r*_*s*_ (88) = 0.24, *p* = 0.01). There was no correlation between emotion regulation and any other content element (understanding: *r*_*s*_ (88) = 0.03, *p* = 0.38; unfairness criticism: *r*_*s*_ (88) = 0.08, *p* = 0.22; questioning of motive: *r*_*s*_ (88) = 0.02, *p* = 0.41; suggestion for usage: *r*_*s*_ (88) = 0.15, *p* = 0.07).

We could affirm that unfair offers in the dictator game decrease subjective happiness. Further, our first hypotheses could be confirmed; writing a message which was transferred to the dictator who made the unfair allocation successfully regulated emotions. Additionally we could demonstrate that successful emotion regulation is related to the extent of emotion expression in the messages.

## Study 2 Methods

92 (*M*_*age*_ = 22.77 years, *SD* = 5.47) female participants took part in study 2. As in study 1 participants were recruited with the software hroot^16^ and the study was conducted at the BonnEconLab at the University of Bonn using z-Tree^20^. The study meets all standards for ethical treatment of human subjects in experiments at the BonnEconLab and was approved by the review board of the department of economics. Written consent was given by all participants according to the Declaration of Helsinki. Study 2 was identical to study 1, but, based on the results of study 1, only the most effective emotion regulation condition (message forwarded) and a control condition were studied. After the three minute waiting or emotion regulation period participants were asked to play the role of the dictator in an additional dictator game with a third person as receiver. Participants were provided with an additional endowment of 10 € and were able to allocate any amount to the receiver. The third person/receivers were invited to the lab at a later time point and received the money the dictators allocated to them.

## Results and Discussion

In order to test whether emotion regulation increases allocations to a third person the allocations between the emotion regulation and control condition were compared. Since the dictator allocations were not normally distributed a Mann-Whitney-U-test for independent samples was used. The results indicate that allocations in the emotion regulation condition (*Mdn* = 4.5) were significantly higher than those in the control condition (*Mdn* = 3; *U* = *987*; *p* = 0.036, *r* = 0.24, 95% C.I. was estimated by using the Hodges Lehmann estimator [−2.00, 0.00]; Fig. 1B right). Furthermore, we observed a positive correlation between happiness ratings after emotion regulation (*Happiness 2*) and the amount participants allocated to the third person (*r*_*s*_ (77) = 0.26, *p* = 0.01). There was no correlation between baseline happiness ratings and the allocations in the dictator game (*r*_*s*_ (77) = 0.11, *p* = 0.16). Thus, baseline differences cannot account for our findings. The results confirm our second hypothesis; participants in the message forwarded condition make higher allocations to a third person compared to the control group.

## Conclusion

In study 1 we could demonstrate that writing a message to the person who made the unfair offer is successful in regulating negative emotions. In comparison to standard emotion regulation strategies, like reappraisal, no training or introduction is needed for writing a message. It is therefore easier to implement in experimental settings. Since we could not find any effect of the neutral picture condition, writing in general can be ruled out as a regulating factor. Although happiness ratings in the message not forwarded condition were not significantly different from the control condition a direct comparison between the two message conditions did not reveal any differences between the two. Forwarding is therefore very likely not a factor driving emotion regulation. However, the correlation between emotional content of the messages and the change in happiness ratings suggests that emotion expression might be a factor driving emotion regulation. Additionally, there are other possible factors, which might drive emotion regulation. Engagement in norm enforcement, perspective taking, punishment via message writing or reflection might for example increase happiness. Further research is needed to determine which process precisley underlies the emotion regulation effect.

Using the dictator game instead of the ultimatum game offered us the possibility to measure emotions without any interaction with the decisions. In line with Xiao and Houser^10^ we demonstrated that writing a message is an effective strategy in regulating negative emotions. Furthermore, we could show that writing in general has no effect.

In study 2, using the emotion regulation strategy, namely writing a message that is forwarded, we could show that people who regulate their emotions make higher offers to a third person, indicating a decrease in generalized negative reciprocity. These results extend the former knowledge about general reciprocity^8^ by providing evidence for negative emotions as a driving factor for generalized negative reciprocity.

We deliberately chose to only measure female participants, since women were shown to show higher emotional reactivity in response to negative stimuli^17^,^18^. Although narrowing the participant pool might have been potentially helpful to find the hypothesized negative emotional response to unfair behavior, at the same time it limits our interpretation. Singer *et al.* have for example shown that male participants showed a greater desire for revenge compared to female participants in response to unfair social partners^21^. Since revenge might also be triggered in our paradigm future studies should first, check whether revenge influences emotion regulations and second, whether there is a gender difference in emotion regulation and subsequent dictator game offers.

To conclude, the results of the two studies show how emotion regulation can influence the affect elicited by unfair allocations of others and thereby interrupt the chain of negative generalized reciprocity. These insights help to further our understanding of social interactions and may help to control the spread of negative reciprocity.

## Additional Information

**How to cite this article**: Strang, S. *et al.* Generalized Negative Reciprocity in the Dictator Game – How to Interrupt the Chain of Unfairness. *Sci. Rep.* **6**, 22316; doi: 10.1038/srep22316 (2016).

## Supplementary Material

Supplementary Information

## References

[b1] Gurven M. To give and to give not: The behavioral ecology of human food transfers. Behav. Brain Sci. 27, 543–583 (2004).

[b2] Gächter S. & Herrmann B. Reciprocity, culture and human cooperation: previous insights and a new cross-cultural experiment. Philos. Trans. R. Soc. 364, 791–806 (2009). 10.1098/rstb.2008.0275 PMC2689715 19073476

[b3] Pillutla M. & Murnighan J. Unfairness, Anger, and Spite: Emotional Rejections of Ultimatum Offers. Organ. Behav. Hum. Decis. Process. 68, 208–224 (1996).

[b4] Sanfey A., Rilling J., Aronson J., Nystrom L. & Cohen J. The neural basis of economic decision-making in the Ultimatum Game. Science. 300, 1755–1758 (2003). 12805551 10.1126/science.1082976

[b5] Van’t Wout M., Kahn R., Sanfey A. & Aleman A. Affective state and decision-making in the Ultimatum Game. Exp. brain Res. 169, 564–568 (2006). 16489438 10.1007/s00221-006-0346-5

[b6] Falk A. & Fischbacher U. A theory of reciprocity. Games Econ. Behav. 54, 293–315 (2006).

[b7] Gintis H. Strong reciprocity and human sociality. J. Theor. Biol. 206, 169–179 (2000). 10966755 10.1006/jtbi.2000.2111

[b8] Gray K., Ward A. F. & Norton M. I. Paying it forward: generalized reciprocity and the limits of generosity. J. Exp. Psychol. Gen. 143, 247–54 (2014). 23244034 10.1037/a0031047

[b9] Nowak M. a & Sigmund K. Evolution of indirect reciprocity by image scoring. Nature 393, 573–577 (1998). 9634232 10.1038/31225

[b10] Xiao E. & Houser D. Emotion expression in human punishment behavior. Proc. Natl. Acad. Sci. USA 102, 7398–401 (2005). 15878990 10.1073/pnas.0502399102 PMC1129129

[b11] Ochsner K. & Gross J. The cognitive control of emotion. Trends Cogn. Sci. 9, 242–249 (2005). 15866151 10.1016/j.tics.2005.03.010

[b12] Goldin P., McRae K., Ramel W. & Gross J. The neural bases of emotion regulation: reappraisal and suppression of negative emotion. Biol. Psychiatry 63, 577–586 (2008). 17888411 10.1016/j.biopsych.2007.05.031 PMC2483789

[b13] Van’t Wout M., Chang L. J. & Sanfey A. G. The influence of emotion regulation on social interactive decision-making. Emotion 10, 815–821 (2010). 21171756 10.1037/a0020069 PMC3057682

[b14] Grecucci A., Giorgetta C., Van’t Wout M., Bonini N. & Sanfey A. Reappraising the ultimatum: an fMRI study of emotion regulation and decision making. Cereb. cortex 23, 399–410 (2013). 22368088 10.1093/cercor/bhs028

[b15] Bradley M. & Lang P. J. Measuring Emotion: The Self-Assessment Manikin And The Semantic Differential. J. Behav. Ther. Exp. Psychiatry 25, (1994). 10.1016/0005-7916(94)90063-9 7962581

[b16] Bock O., Nicklisch A. & Baetge I. hroot: Hamburg registration and organization online tool. WiSo-HH Work. Pap. Ser. 1, (2012).

[b17] Bradley M. M., Codispoti M., Sabatinelli D. & Lang P. J. Emotion and motivation II: sex differences in picture processing. Emotion 1, 300–319 (2001). 12934688

[b18] Grossman M. & Wood W. Sex differences in emotional intensity. J. Pers. Soc. Psychol. 65, 1010–1022 (1993). 8246109 10.1037//0022-3514.65.5.1010

[b19] Lang P., Bradley M. & Cuthbert B. In Electric Power Systems Research (University of Florida, 2008).

[b20] Fischbacher U. z-Tree: Zurich toolbox for ready-made economic experiments. Exp. Econ. 10, 171–178 (2007).

[b21] Singer T. *et al.* Empathic neural responses are modulated by the perceived fairness of others. Nature 439, 466–9 (2006). 16421576 10.1038/nature04271 PMC2636868

