# Supplementary material for: Generalized Negative Reciprocity in the Dictator Game – How to Interrupt the Chain of Unfairness
Source: Sci Rep. 2016 Feb 29;6:22316. doi: 10.1038/srep22316 (PMC4770415; doi:10.1038/srep22316)
Supplement: Supplementary Information [file srep22316-s1.pdf]

Supplementary Information

Generalized Negative Reciprocity in the Dictator Game – How to Interrupt the Chain of Unfairness

Sabrina Strang, Xenia Grote, Katarina Kuss, Soyoung Q Park and Bernd Weber

S1. Instructions online experiment

Thanks a lot for your participation in our online-questionnaire. The questionnaire will take approximately 5 min.

When you finished the questionnaire, you can take part in a prize draw to win one of five Amazon-vouchers with a value of 10€ each. For this purpose please indicate your mail address at the end of the questionnaire. Your mail address will be saved separated from your answers during the questionnaire and will be used for the prize draw only. The five winners of the prize draw will be informed via mail.

To start the questionnaire please push >>

Hereafter you will be asked to evaluate 15 messages according to five different aspects. This messages were written after receiving an unfair money allocation and are directed to the person who made the unfair allocation. Please indicate whether a certain message contains one or more of the aspects.

Please read the subsequent clarification of the five aspects carefully. During the evaluation you will only see the general terms of the given aspects and not the additional clarification.

Expression of emotions

Please chose expression of emotion in case the message contains any emotional expression of the person who wrote the message.

Understanding

Please chose understanding in case the message contains any understanding of the behavior of the person how made the unfair money allocation.

Unfairness criticism

Please chose unfairness criticism in case the message contains any critique concerning for example unfair behavior, egoism, violation of social norms etc.

Questioning of motive

Please chose questioning of motive in case the message contains any question concerning the reason for the unfair behavior or other questions concerning the unfair money allocation.

Suggestion for usage

Please chose suggestion for usage in case the message contains any information about the usage of the money.

Please perform the evaluation conscientiously. Read all messages carefully and chose those aspects which are, according to your opinion, contained in the corresponding message. Every message can contain more than one aspect, multiple answers are therefore possible.

After reading the above clarification carefully, please push >>

This is the end of the online questionnaire.
Thank you very much for your participation.

Please indicate your mail address below to take part in the prize draw.

S2. Analysis fair allocations

We tested whether there is a difference in the change of happiness ratings between participants who were treated fairly and those who were treated unfairly in both studies. Therefore, we compared Happiness 1 minus baseline Happiness ratings between the two groups in each study. We found significant differences in the change of the ratings (study 1: $p < .001$; $U = 237.00$; study 2: $p < .001$; $U = 926.00$). While participants who were treated fairly reported an increase in happiness (study 1: Mdn = 1; study 2: Mdn = 1), those who were treated unfairly reported a decrease in happiness ratings (study 1: Mdn = -2; study 2: Mdn = -2).

In order to test whether there is a difference in emotion regulation in study 1 between participants who were treated fairly and those who were treated unfairly we performed the same ANCOVA as for unfairly treated participants an could not find any significant difference between emotion regulation conditions in the change of happiness rating between conditions ($p = .341$, $F = 1.165$).

In study 2 we compared dictator allocations of participants who were treated fairly with those who were treated unfairly in order to assure that we can replicate the generalized reciprocity phenomenon (Gray et al., 2014; higher allocations of participants treated fairly compared to those of participants who were treated unfairly). Participants who were treated fairly made significant higher allocations (Mdn = 5) in the dictator game than those who were treated unfairly (Mdn = 3; $p < 0.01$; $U = 709.50$; $r = .29$). Our data is thus in line with previous data on generalized reciprocity (Gray et al., 2014).

However, since only 17% (in study 1 N=8-12 and study 2 N=12 per condition) of the participants were treated fairly, these results above should be interpreted carefully.
